# Associations between HIV testing and multilevel stigmas among gay men and other men who have sex with men in nine urban centers across the United States

**DOI:** 10.1186/s12913-022-08572-4

**Published:** 2022-09-20

**Authors:** Kate E. Dibble, Sarah M. Murray, John Mark Wiginton, Jessica L. Maksut, Carrie E. Lyons, Rohin Aggarwal, Jura L. Augustinavicius, Alia Al-Tayyib, Ekow Kwa Sey, Yingbo Ma, Colin Flynn, Danielle German, Emily Higgins, Bridget J. Anderson, Timothy W. Menza, E. Roberto Orellana, Anna B. Flynn, Paige Padgett Wermuth, Jennifer Kienzle, Garrett Shields, Stefan D. Baral

**Affiliations:** 1 Department of Epidemiology, Johns Hopkins Bloomberg School of Public Health, 615 N. Wolfe Street, Baltimore, MD 21205 USA; 2 Department of Mental Health, Johns Hopkins Bloomberg School of Public Health, Baltimore, MD 21205 USA; 3 Department of Health, Behavior and Society, Johns Hopkins Bloomberg School of Public Health, Baltimore, MD 21205 USA; 4 Denver Public Health & Hospital Authority, Denver, CO 80204 USA; 5 Los Angeles County Department of Public Health, Los Angeles, CA 90012 USA; 6 Center for HIV Surveillance, Epidemiology, & Evaluation, Maryland Department of Health, Baltimore, MD 21202 USA; 7 Division of HIV and STI Programs, Michigan Department of Health and Human Services (MDHHS), Lansing, MI 48909 USA; 8 Bureau of Communicable Disease Control, New York State Department of Health, Albany, NY 12237 USA; 9 HIV Data and Analysis Program, Oregon Health Authority, Portland, OR 97232 USA; 10 Regional Research Institute, Portland State University, Portland, OR 97201 USA; 11 School of Social Work, University of Washington, Seattle, WA 98105 USA; 12 Surveillance & Federal Reporting Section, Maternal, Child, & Adolescent Health Division, California Department of Health,, San Francisco, CA 94102 USA; 13 Division of Management Policy & Community Health (MPACH), The University of Texas Health Science Center at Houston (UTHealth) School of Public Health, Houston, TX 77030 USA; 14 Division of Disease Prevention, Virginia Department of Health, HIV Surveillance, Richmond, VA 23219 USA

**Keywords:** HIV testing, Stigma, HIV criminalization laws, Men who have sex with men, United States

## Abstract

**Background:**

Complex manifestation of stigma across personal, community, and structural levels and their effect on HIV outcomes are less understood than effects in isolation. Yet, multilevel approaches that jointly assesses HIV criminalization and personal sexual behavior stigma in relation to HIV testing have not been widely employed or have only focused on specific subpopulations. The current study assesses the association of three types of MSM-related sexual behavior-related stigma (family, healthcare, general social stigma) measured at both individual and site levels and the presence/absence of laws criminalizing HIV transmission with HIV testing behaviors to inform HIV surveillance and prevention efforts among HIV-negative MSM in a holistic and integrated way.

**Methods:**

We included nine National HIV Behavioral Surveillance (NHBS) 2017 sites: Baltimore, MD; Denver, CO; Detroit, MI; Houston, TX; Long Island/Nassau-Suffolk, NY; Los Angeles, CA; Portland, OR; San Diego, CA; and Virginia Beach and Norfolk, VA. Multivariable generalized hierarchical linear modeling was used to examine how sexual behavior stigmas (stigma from family, anticipated healthcare stigma, general social stigma) measured at the individual and site levels and state HIV criminalization legislation (no, HIV-specific, or sentence-enhancement laws) were associated with past-year HIV testing behaviors across sites (*n* = 3,278).

**Results:**

The majority of MSM across sites were tested for HIV in the past two years (*n* = 2,909, 95.4%) with the average number of times tested ranging from 1.79 (*SD* = 3.11) in Portland, OR to 4.95 (*SD* = 4.35) in Los Angeles, CA. In unadjusted models, there was a significant positive relationship between stigma from family and being tested for HIV in the past two years. Site-level HIV-specific criminalization laws were associated with an approximate 5% reduction in the prevalence of receiving any HIV test in the past two years after individual level stigma and sociodemographic covariate adjustments (PR = 0.94, 95% CI, 0.90–0.99).

**Conclusions:**

Structural barriers faced by MSM persist and ending the HIV epidemic in the US requires a supportive legal environment to ensure effective engagement in HIV services among MSM. Home-based solutions, such as self-testing, used to deliver HIV testing may be particularly important in punitive settings while legal change is advocated for on the community and state levels.

**Supplementary Information:**

The online version contains supplementary material available at 10.1186/s12913-022-08572-4.

## Background

The HIV epidemic in the United States (US) disproportionately impacts cisgender gay, bisexual, and other men who have sex with men (MSM). Male-to-male sexual contact accounted for 66% of incident HIV infections in 2018 [1], and one in every six MSM living with HIV remains unaware of their HIV status [1]. Despite CDC recommendations [2, 3] to conduct annual HIV screening for sexually active MSM, more than one in four (27%) MSM reported not having tested in the last year [4]. Despite having numerous successful HIV testing modalities available in the US (e.g., community- or facility-based, peer- or partner-distributed, online-mail, etc.), some approaches have been shown to be more effective than others in improving testing uptake (e.g., online-mail, self-testing kits distributed at medical facilities) compared to traditional facility-based testing [5]. Increased uptake and frequency of HIV testing can improve linkage to HIV treatment and aid efforts to prevent onward transmission [6].

Personal experiences of perceived, internalized, anticipated, and enacted stigma have been demonstrated to serve as a barrier to HIV testing [7–9], and growing evidence suggests that structural-level stigma may discourage HIV disclosure and testing [10–12]. For instance, a US national sample of HIV-negative MSM found that a less stigmatizing political climate was shown to be positively associated with MSM’s comfortability discussing sexual health with a primary care provider [13], which in turn has been linked to lowered HIV risk via access to pre- and post-exposure prophylaxis [14]. Currently as of 2022, 29 states in the US have statutory laws that criminalize sexual behavior that results in HIV transmission compared to only nine states in 2014. Since the time the NHBS data were collected, an additional four states (California, Missouri, Nevada, Virginia) have amended their legislation, requiring “intention to transmit” or including these regulations among those for disease control instead of criminalization [10]. HIV criminalization imposes penalties on the alleged, perceived, or potential of exposing persons to HIV through nondisclosure of known HIV-positive status prior to sexual contact or non-intentional HIV transmission [10]. Despite the presence of HIV criminalization legislation, cities/states differ in ways pertinent to HIV testing and related legislative awareness [15]. Across several past studies, MSM who lived in states with HIV criminalization laws and were HIV-negative or with an unknown HIV status reported increased sexual risk behavior and decreased HIV testing compared to those who lived in states without these laws [11, 16–21].

Complex manifestations of stigma across personal, community, and structural levels and their effect on HIV outcomes are less understood than effects of either in isolation [7]. HIV testing and stigma are likely to interact in multifaceted ways, and appropriate design and implementation of interventions in diverse US sociopolitical contexts requires further exploration of the joint contribution of sociodemographic, environmental, and personal factors at various levels (personal, city/site, state). Yet, multilevel approaches that jointly assesses HIV criminalization and personal sexual behavior stigma in relation to HIV testing have not been widely employed or have only focused on specific subpopulations [3, 8, 22, 23]. Although there have been similar studies focusing on the impact of multilevel stigmas and HIV testing behaviors in Central Asia and sub-Saharan Africa [24–26], the current study provides novelty in analyzing several types of sexual behavior stigma on both individual and site levels within US metropolitan areas. The current study assesses the association of three types of MSM-related sexual behavior-related stigma (family, healthcare, general social stigma) measured at the personal and site level and the presence/absence of laws criminalizing HIV transmission with HIV testing behaviors to inform HIV surveillance and prevention efforts among HIV-negative MSM in a holistic and integrated way. Identifying stigma-related correlates of HIV testing using a multilevel perspective can inform efforts to boost HIV testing at a community level.

## Methods

NHBS collects data on an annual rotating basis among three groups considered “at high risk” for HIV infection [27]. Each of the 22 NHBS sites in the 2017 cycle focused on MSM were provided the option to include a module of 13 stigma-related questions into their local questions; 9 out of 22 sites included this module and shared their data: 1) Baltimore, Maryland, 2) Denver, Colorado, 3) Detroit, Michigan, 4) Houston, Texas, 5) Long Island/Nassau-Suffolk, New York, 6) Los Angeles, California, 7) Portland, Oregon, 8) San Diego, California, and 9) Virginia Beach and Norfolk, Virginia.

### Study sample & procedure

NHBS used venue-based, time-location sampling to randomly select specific day/time periods for recruitment of MSM at numerous venues (e.g., bars, clubs, organizations, street locations, etc.). Each site identified their own venues attended by MSM as detailed elsewhere [1, 28]. NHBS eligibility criteria included aged 18 years or older; current residence in an NHBS-defined geographic location; no previous participation in NHBS during the current cycle; ability to complete the survey in either English or Spanish; ability to provide written informed consent [1]; assigned male sex at birth; current male gender identity; and lifetime oral or anal sex with another man.

Written consent was obtained from participants prior to beginning study procedures. A trained interviewer collected demographic information and data on behavioral risks for HIV infection, histories of HIV and STI testing, and use of other HIV preventive resources via an anonymous computer-assisted personal interview (CAPI) [29]. Participants received compensation for survey completion ($20–30 depending on site) and completion of anonymous HIV, STI, and/or hepatitis tests ($10–50 depending on site) [27]. For this secondary data analysis, we restricted our sample to those who tested negative for HIV.

Each site obtained approval to administer the NHBS surveys from the institutional review board (IRBs) at the departments of health within their metropolitan statistical area or deferred to the Johns Hopkins Bloomberg School of Public Health (JHSPH) IRB, the IRB of record (IRB#00007006). All research procedures were conducted in accordance with the JHSPH IRB and the Declaration of Helsinki.

### Measures

#### Demographic covariates

Demographic information was collected via self-report survey and included: age (continuous, assessed via date of birth), sexual identity, education, and race, which were dummy coded. For example, race was dummy coded so that each race was represented by its own dichotomous variable, except for Asian and Native Hawaiian/Other Pacific Islander populations, which were not included due to invariability and insufficient group size in relation to the outcome.

#### Sexual behavior stigma exposure

Sexual behavior stigma items were originally developed by applying the previously-published modified social ecological model (MSEM) [30] to studies of HIV risks among MSM, identifying barriers in social capital and community services within numerous populations [21–23]. The factor structure and internal reliability have been assessed previously these data [31], with the nine included items found to load onto three factors: 1) stigma from family (2 items; possible range: 0–2; α = 0.64–0.75), 2) anticipated healthcare stigma (2 items; possible range: 0–2; α = 0.61–0.80), and 3) general social stigma (5 items; possible range: 0–5; α = 0.62–0.68) [31]. Item examples are listed in Appendix Table 1. Each of the 9-items had three response options (no; yes, in the past year; yes, but not in the past year), but for analytic purposes due to invariability, item responses were dichotomized by collapsing the affirmative responses as done in previous analyses [31]. Average participant sexual behavior stigma scale scores by site were also included as a second level (site-level) fixed effect.

#### HIV criminalization exposure

Presence or absence of state HIV criminalization laws was included as a site-level fixed effect. Following CDC categorization of laws [10] that directly involve HIV transmission, we categorized states as having HIV criminalization laws if they either had: 1) HIV-specific criminal laws that criminalized behaviors that can potentially expose other persons to HIV (affirmative legislation); or 2) Sentence enhancement laws specific to HIV that do not criminalize behavior of transmission but increase sentence length when an personal commits specific crimes (i.e., sexual crimes, etc.) while infected with HIV. If a state fell into neither category, we considered the state to not have HIV criminalization legislation [10, 32]. The following NHBS sites had HIV-specific criminalization in place in their state during 2017: Baltimore, Maryland; Detroit, Michigan; Los Angeles, California; San Diego, California; and Virginia Beach and Norfolk, Virginia (See Appendix Table 2 for more details).

#### HIV testing

HIV testing was assessed as whether someone had tested for HIV in the past two years (yes/no), and among those who had tested, the number of times an personal had tested in the past two years. Due to a non-normal distribution for the testing frequency, this count variable of number of times tested in the past two years was log transformed and ranged from zero to 30.

### Statistical analysis

We conducted hierarchical generalized linear modeling (HGLM) to assess associations of stigma factors at different levels of the social ecology (i.e., personal and site level) with HIV testing behavior, with the model intercept allowed to vary randomly by NHBS site [33, 34]. Analyses were conducted separately for each HIV testing outcome: the binary outcome of any versus no HIV test in the past two years; and the continuous outcome of log number of HIV tests received in the past two years among those with any test during that period. Two models were conducted overall, each including the three sexual behavior stigma factors and average factor scores by site (see Appendix 3 for conceptual diagram). For the outcome of any HIV test in the past two years, a modified Poisson regression was run using Poisson family and log link with the meglm command (via Stata version 16 [35], as this outcome was very common [36, 37]. For the log number of times an HIV test was received among those who tested in the past two years, generalized linear model (GLM) was fit with family specified as Gaussian.

First, a model was run with the inclusion of a random intercept for site and only personal participant-level average sexual behavior stigma subscales (within-site effect of stigma) (Model 1). We re-ran this model including potential confounders selected based on existing evidence and whether or not potential confounders were statistically significantly associated with any of the sexual behavior stigma subscales and either of the two HIV testing variables using Poisson log-linear and Poisson log-linear count regressions (Model 2) [6, 12, 15, 16, 18, 21]. Thus, we included the same covariates across all models as covariate analyses, combined with past literature, were similar across outcomes. Lastly, we added site-level predictors to Model 2 to generate coefficient estimates for personal reports of stigma while accounting for HIV criminalization laws and the average stigma subscale scores by site (between-site effect of stigma) (Model 3). Preliminary, exploratory model analyses were conducted in SPSS© Version 27 [38] and final GLM models were run in STATA Release 16 [35].

## Results

### Participant characteristics

A total of 3,278 HIV-negative MSM were included across the 9 sites. In Table 1, demographic characteristics are presented for the total sample and by whether someone reported ever being tested for HIV. Most participants were non-Hispanic (*n* = 2475; 75.6%), white (*n* = 1670; 50.9%) or identified as homosexual or gay (*n* = 2507; 76.5%) with a mean age of 35.1 years (*SD* = 12.0; Range: 18 to 85 years) (See Table 3). Most reported being tested for HIV within the past two years (*n* = 2922, 89.1%). The mean number of times tested in the past two years among those who ever tested ranged 1.79 (*SD* = 3.11) in Portland, Oregon to 4.95 (*SD* = 4.35) in Los Angeles, California. The range of sexual behavior stigma-related experiences varied, such as ever being forced to have sex when the individual did not want to and believed it was because they are MSM (*n* = 273, 8.3%) to ever being verbally harassed because they are MSM (*n* = 1,408, 43.0%).

**Table 1:** Participant demographic characteristics – National HIV Behavioral Surveillance, 9 U.S. sites, 2017

**Characteristics**	**Total sample** **(*N* = 3278)**	Tested within the past two years (*n*= 2922)	Did not test within the past two years (*n*= 356)
	**No. (%)**	**No. (%)**	**No. (%)**
**Race/ethnicity**			
American Indian or AN^a^	108 (3.3)	98 (3.4)	10 (2.8)
Asian	77 (2.3)	65 (2.2)	12 (3.4)
Black/African American	931 (28.4)	876 (30.0)	55 (15.4)
Hispanic/Latino^b^	800 (24.4)	722 (24.7)	78 (21.9)
Native Hawaiian or OPI^c^	35 (1.1)	33 (1.1)	2 (0.6)
White	1670 (50.9)	1447 (49.5)	223 (62.6)
Multiple races	271 (8.3)	230 (7.9)	41 (11.5)
Missing	186 (5.7)	173 (5.9)	13 (3.7)
**Sexual Identity**			
Gay, same gender loving, or homosexual	2507 (76.5)	2238 (76.6)	269 (75.6)
Bisexual	646 (19.7)	571 (19.5)	75 (21.1)
Straight or heterosexual	57 (1.7)	49 (1.7)	8 (2.2)
Missing	68 (2.0)	64 (2.2)	4 (1.1)
**Employment**			
Employed full- or part-time	2679 (81.7)	2374 (81.2)	305 (85.7)
Unemployed^d^	490 (14.9)	447 (15.3)	43 (12.1)
Did not report employment	109 (3.3)	101 (3.5)	8 (2.2)
**Education**			
High school/GED or below	766 (23.4)	692 (23.7)	74 (20.8)
Some college or above	2483 (75.7)	2202 (75.4)	281 (78.9)
Missing	29 (0.9)	28 (1.0)	1 (0.3)
**NHBS Site**			
Baltimore, MD	354 (10.8)	335 (11.5)	19 (5.3)
Denver, CO	455 (13.9)	450 (15.4)	5 (1.4)
Detroit, MI	377 (11.5)	369 (12.6)	8 (2.2)
Houston, TX	420 (12.8)	324 (11.1)	96 (27.0)
Long Island/Nassau-Suffolk, NY	150 (4.6)	124 (4.2)	26 (7.3)
Los Angeles, CA	442 (13.5)	442 (15.1)	0 (0.0)
Portland, OR	355 (10.8)	162 (5.5)	193 (54.2)
San Diego, CA	436 (13.3)	435 (14.9)	1 (0.3)
Virginia Beach & Norfolk, VA	289 (8.8)	281 (9.6)	8 (2.2)
	**Mean (*SD*)**	**Mean (*SD*)**	**Mean (*SD*)**
**Participant Age**	35.1 (12.0)	35.1 (12.1)	35.9 (11.6)
**Stigma **			
Stigma from family	0.36 (0.41)	0.35 (0.41)	0.39 (0.43)
Anticipated healthcare stigma	0.10 (0.26)	0.10 (0.27)	0.14 (0.31)
General social stigma	0.24 (0.27)	0.23 (0.27)	0.34 (0.30)

^a^AN = Alaska Native

^b ^Hispanics/Latinos can be of any race

^c ^OPI = Other Pacific Islander

^d^ The “unemployed” category includes unemployed, unable to work for health reasons, retired, or student response options

### Stigma, HIV testing, and HIV criminalization

Results from the HGLM models with whether a participant received any HIV test in the past two years as the outcome are presented in Table 2. In Model 1, a one unit increase in total stigma from family was associated with a statistically significant increase in the prevalence of having received an HIV test in the past two years (prevalence ratio [PR] = 1.022, 95% CI, 1.004–1.040), but neither anticipated healthcare stigma (*p* = 0.122) nor general social stigma (*p* = 0.466) was significantly associated with this outcome. Adjusting for covariates in Model 2, stigma from family was no longer statistically significantly associated with having tested for HIV in the past 2 years (PR = 1.016, 95% CI, 0.995–1.036). When adding site level variables in Model 3, the presence of HIV-specific criminalization and/or sentence enhancement laws/statutes was associated with an approximate 5% reduction in the prevalence of receiving an HIV test (PR = 0.948, 95% CI, 0.903–0.996).

**Table 2:** Generalized hierarchical linear model (HLM) analyses examining the effects of personal- and area/site-level on whether someone has HIV tested in the past 2 years

		**Model 1** **(*N* = 2,977)**	**Model 2** **(*N* = 2,846)**	**Model 3** **(*N* = 2,846)**
		Prevalence ratio (*SE*), *p*-value	Prevalence ratio (*SE*), *p*-value	Prevalence ratio (*SE*), *p*-value
**Personal-level stigma and discrimination**	Stigma from family	1.022 (0.009), ***p* = 0.018**	1.016 (0.010), *p* = 0.133	1.014 (0.011), *p* = 0.186
	Anticipated healthcare stigma	0.975 (0.016), *p* = 0.122	0.976 (0.020), *p* = 0.242	0.977 (0.020), *p* = 0.255
	General social stigma	1.004 (0.006), *p* = 0.466	1.007 (0.006), *p* = 0.238	1.005 (0.005), *p* = 0.370
**Covariates**	Age in years		0.997 (0.002), *p* = 0.056	0.997 (0.002), *p* = 0.068
	Education		0.999 (0.018), *p* = 0.944	0.996 (0.015), *p* = 0.767
	Sexual orientation		1.006 (0.076), *p* = 0.942	1.005 (0.081), *p* = 0.947
**Site-level stigma and discrimination**	Stigma from family site average			1.102 (0.232), *p* = 0.645
	Anticipated healthcare stigma site average			0.956 (0.620), *p* = 0.945
	General social stigma site average			1.043 (0.096), *p* = 0.646
	Criminalization			0.948 (0.024), ***p* = 0.035**

*Note.* Bold font indicates factors remained significantly related to the outcome controlling for all included personal-level, demographic covariates; Age, stigma from family, anticipated healthcare stigma, general social stigma, and site average predictors are continuous; Education (0 = high school graduate/GED or less; 1 = some college or above); Sexual orientation (0 = heterosexual or “straight”; 1 = homosexual, gay, lesbian, bisexual, other); HIV criminalization (0 = no HIV-specific criminalization laws; 1 = HIV-specific criminalization and/or sentence enhancement laws/statues)

Results of the multilevel linear regression analyses assessing the log number of times tested among those who had tested in the past two years are presented in Table 3. A one unit increase in total stigma from family score was associated with a small but statistically significant increase in the log number of tests received in the past two years (β = 0.022, 95% CI, 0.005–0.039). This effect was attenuated and no longer significant after adjustment in Model 2 (β = 0.015, 95% CI, -0.003–0.033). In Model 3, average general social stigma score in a site was the only stigma related variable that remained statistically significant, with a one-unit increase associated with a 0.261-point increase (95% CI, 0.042–0.479) in log number of tests received in the past two years.

**Table 3:** Generalized hierarchical linear model (HLM) analyses examining the effects of personal- and area/site-level in the log number of times HIV tested in the past 2 years among those with any test

		**Model 1** **(*N* = 2,528)**	**Model 2** **(*N* = 2,184)**	**Model 3** **(*N* = 2,184)**
		β (*SE*), *p*-value	β (*SE*), *p*-value	β (*SE*), *p*-value
**Personal-level stigma and discrimination**	Stigma from family	0.022 (0.008) ***p* = 0.012**	0.015 (0.009), *p* = 0.105	0.015 (0.009), *p* = 0.105
	Anticipated healthcare stigma	0.003 (0.012), *p* = 0.784	0.011 (0.013), *p* = 0.409	0.011 (0.013), *p* = 0.413
	General social stigma	0.009 (0.007), *p* = 0.164	0.011 (0.007), *p* = 0.132	0.011 (0.007), *p* = 0.145
**Covariates**	Age in years		-0.003 (0.001), ***p* < 0.001**	-0.003 (0.001), ***p* < 0.001**
	Education		0.038 (0.17), ***p* = 0.024**	0.039 (0.017), ***p* = 0.022**
	Sexual orientation		-0.070 (0.066), *p* = 0.293	-0.070 (0.066), *p* = 0.288
**Site-level stigma and discrimination**	Stigma from family site average			-0.431 (0.282), *p* = 0.127
	Anticipated healthcare stigma site average			0.928 (0.682), *p* = 0.173
	General social stigma site average			0.261 (0.112), ***p* = 0.020**
	Criminalization			-0.067 (0.035), *p* = 0.059

*Note.* Bold font indicates factors remained significantly related to the outcome controlling for all included personal-level, demographic covariates; Age, stigma from family, anticipated healthcare stigma, general social stigma, and site average predictors are continuous; Education (0 = high school graduate/GED or less; 1 = some college or above); Sexual orientation (0 = heterosexual or “straight”; 1 = homosexual, gay, lesbian, bisexual, other); HIV criminalization (0 = no HIV-specific criminalization laws; 1 = HIV-specific criminalization and/or sentence enhancement laws/statues)

## Discussion

The purpose of this study was to characterize the multilevel association of sexual behavior stigma experiences at the personal, community, and structural state level policies with HIV testing practices. The vast majority of the sample reported testing for HIV at least once in the past two years, higher than what has been found in previous literature but consistent with an increasing trend among MSM in the US [39] and perhaps in part explained by the overall high level of education obtained in this sample [40–42]. Those who had tested versus not in the past two years were less likely to endorse stigma from family, anticipated healthcare stigma, and general social stigma consistent with prior literature [22, 43], but these differences were not significant in our study, as this association became nonsignificant after adjustment of potential confounders. We were unable to limit the assessment of stigma to recent (past 12 months) experiences due to low reported prevalence. It is possible that asking about lifetime experiences attenuated associations by capturing experiences with little temporal proximity to HIV testing behaviors or that we did not assess forms of stigma that may have been related to testing (e.g., internalized stigma).

HIV criminalization laws were significantly negatively associated with HIV testing, a possible indication of the more consequential role that structural stigma may play in shaping HIV testing behaviors among MSM. Sexual behavior stigmas may have a more direct impact on the uptake of HIV testing accounting for the perceived consequences of HIV criminalization. This finding contributes to limited existing literature from the US and Canada that has linked HIV criminalization laws to decreased HIV testing [11, 16, 18, 44, 45], and supports long-held concerns on the impact of enshrining HIV stigma into law [15, 46–49]. Being guided by successful advocacy work by persons living with (PLHIV) and others thus far, in the US and elsewhere (e.g., HIV Justice Network, Global Network of People Living with HIV), will be integral moving forward [46].

Some have argued that people may intentionally refrain from testing out of fear of legal repercussions for committing an HIV-related offense (e.g., non-disclosure of positive status prior to sex) [47, 50]. This argument assumes that people are aware of and understand HIV criminalization laws in their state or district, though this knowledge has been found to be relatively uncommon [21, 51–53] unless media attention has been brought to the application of such laws [16, 18]. Future research should begin to examine the pathways through which policy-level structural stigma may indirectly shape HIV risk behaviors. In addition, we did not assess cross-level interactions between HIV criminalization and personal-level stigma on account of a lack of significant findings at the personal level within this sample. However, this remains an important future direction of research to understand how sexual behavior and associated stigmas across socioecological levels may interact to impact HIV testing and other sexual health outcomes. In particular, an important consideration in examining HIV criminalization laws, in combination with sexual behavior stigma, is how these laws may impact MSM differently than non-MSM, pointing to a need to apply an intersectional lens to policy analysis [54, 55]. Future research should also investigate the role of race/ethnicity and other socioeconomic factors (income, education, residential locale, etc.), most namely as mediators, on the multilevel relationship between sexual behavior stigmas and HIV testing behaviors in combination with changes in HIV criminalization and policy present across the US.

The finding that general social stigma experiences aggregated at the personal level were positively associated with frequency of testing is unlike much of the existing literature reporting on the negative impact of stigma on HIV testing among MSM [56–59]. This finding may be explained by higher levels of general social stigma among certain NHBS sites [60, 61]. In these areas, MSM may be demonstrating resiliency and attempting to protect their health by testing more often [62] in response to perceived or actual increased risk of HIV infection, based on the state of their areas’ respective HIV epidemics. General social stigma may be higher in areas where MSM experience a higher HIV prevalence simply based on exposure, the general community’s level of awareness of links between MSM communities and HIV, level of outness, and potentially larger MSM networks (where MSM may be aware of many instances of MSM in their communities being stigmatized). Our sample is limited to MSM who opted-in to HIV testing at the time of survey administration, and it is therefore possible that our sample is comprised of MSM who test frequently and who may generally be more open about their sexuality and sexual health, which may incur more sexual behavior stigma. The sites with higher general social stigma could have HIV programs that work to develop better testing infrastructure with regard to non-traditional approaches that do not require visits to health centers, community-based, or self-testing [63]; these testing strategies might be in response to high levels of stigma and have been successful in increasing testing frequency.

In response to current and past literature findings, future research should increasingly focus on community level or statewide interventions to address several multifactorial changes that would increase HIV testing behavior among MSM across the US. Across sociopolitical levels (policy, community, interpersonal, individual), stigma mitigation programs have been found to feasibly reduce the experience of anticipated and enacted stigma among MSM in the US through self-acceptance, socialization and partnership, knowledge-sharing, introspection, and self-reflection, among others [64, 65]. More research is needed, however, to determine feasibility among other historically marginalized communities in addition to communities of gay men and other MSM. In addition to HIV decriminalization, decentralizing HIV testing can further mitigate the effects of structural stigmas. Past literature has shown that US-based gay men and other MSM favored self-testing web-based tools with mail delivery of HIV tests and receiving self-tests via facility distribution over centralized facilities that offer in-person testing [5]. Self-testing options, like those noted in this discussion, are convenient and confidential [5], even when receiving test results. Future interventions involving HIV self-testing should ensure that other marginalized groups, such as those without internet access, are able to access self-testing options, even in rural settings. Relatedly, recent research and the World Health Organization (WHO) has suggested linking HIV testing and antiretroviral therapy (ART) to improve early HIV diagnosis and treatment initiation as well as to reduce the costs of a lapse in beginning treatment [65–68]. 

### Limitations

The nine sites included in the current study are metropolitan and primarily coastal areas, limiting generalizability of our findings to interior and rural areas. Generalizability may also be limited to those who frequent venues from which NHBS recruited (e.g., bars, clubs, organizations, street locations), which may represent a more “out” population of MSM which may be fundamentally different in terms of stigma experiences than the full population of MSM. Thus, this population may be more motivated to get an HIV test as opposed to those who do not test due to perceived stigma from providers or internalized stigma, which was not analyzed in the current study. Participants in the current study, therefore, may experience sexual behavior stigmas differently than those who did not visit the facilities in which they were recruited. Perhaps one reason for this may be due to the increase of self-testing across the US, as stigma may affect MSM using self-testing methods less. This concept was not accounted for in our analyses. It is also possible that participants were unaware (or aware) of the HIV criminalization legislation present in their state at the time of data collection, which may further affect HIV testing behaviors and perceived stigmas relating to testing. Additionally, since 2014 when HIV criminalization legislation and NHBS data were collected, an additional four states (California, Missouri, Nevada, Virginia) have amended their legislation, requiring “intention to transmit” or including these regulations among those for disease control [10]. Additionally, the majority of the current sample was white, indicating lack of generalizability to minority racial/ethnic groups, whom are at greater risk for experiencing a multitude of stigmas and discrimination.

The cross-sectional design of this study means observed associations between stigma and testing cannot be interpreted as causal. The current study asked about lifetime stigma experiences and past two year testing behaviors, so experiences of stigma may have occurred after testing or as mentioned so long ago that they no longer impact current behavior. Among the current sample, HIV testing was common overall and anticipated healthcare stigma was relatively rarely reported. Thus, invariability between the HIV testing and stigma variables may have limited our ability to observe significant stigma predictions at the personal level. The anticipated healthcare stigma and the family stigma measure both contained only two items, meaning we may have missed important experiences related to these constructs, attenuating associations with testing behaviors.

## Conclusions

HIV criminalization was associated with a decreased uptake of HIV testing among MSM across 9 US metropolitan sites, highlighting the potential role of punitive policies in creating and sustaining barriers for MSM to HIV services. Associations observed between higher average social stigma being reported in an area and higher frequency of testing may be due to the general community’s link between MSM communities and HIV testing, coupled with the level of outness. Structural barriers faced by MSM persist and ending the HIV epidemic in the US requires a supportive legal environment to ensure effective engagement in HIV services among MSM. Technologically-based solutions, such as telemedicine, used to deliver HIV testing may be particularly important in punitive settings while legal change is advocated for on the community and state levels.

## Supplementary Information

**Additional file 1: Appendix Table 1. **Sexual behavior stigma scale items and responses by associated factors.

**Additional file 2: Appendix Table 2. **HIV-specific criminalization laws – National HIV Behavioral Surveillance, 9 U.S. sites, 2017.

**Additional file 3: Appendix 3 Figure.** Conceptual diagram of multivariable generalized hierarchical linear model combining individual and site factors.

## Data Availability

The datasets used and/or analyzed during the current study available from the principal investigator, Dr. Stefan Baral (sbaral@jhu.edu), on reasonable request.

## Abbreviations

CAPI: Computer-assisted personal interview
CDC: Centers for Disease Control and Prevention
CI: Confidence interval
GLM: Generalized linear model
HGLM: Hierarchical generalized linear modeling
HIV: Human immunodeficiency virus
JHSPH: Johns Hopkins Bloomberg School of Public Health
MSM: Men who have sex with men
NHBS: National HIV Behavioral Surveillance
PLHIV: Persons living with HIV
PR: Prevalence ratio
SD: Standard deviation
SPSS: Statistical Program for the Social Sciences
STI: Sexually transmitted infections
US: United States
