# Supplementary material for: Associations between HIV testing and multilevel stigmas among gay men and other men who have sex with men in nine urban centers across the United States
Source: BMC Health Serv Res. 2022 Sep 20;22:1179. doi: 10.1186/s12913-022-08572-4 (PMC9490978; doi:10.1186/s12913-022-08572-4)
Supplement: Supplementary file 1 — Additional file 1: Appendix Table 1. Sexual behavior stigma scale items and responses by associated factors. [file 12913_2022_8572_MOESM1_ESM.docx]

| **Appendix Table 1.** Sexual behavior stigma scale items and responses by associated factors | | |
| --- | --- | --- |
| **Factors** | **Item** | **Item Description** |
| **Stigma from Family** | 1 | Have you ever felt excluded from family activities because you have sex with men? |
|  | 2 | Have you ever felt that family members have made discriminatory remarks or gossiped about you because you have sex with men? |
| **Anticipated Healthcare Stigma** | 4 | Have you ever felt afraid to go to health care services because you worry someone may learn you have sex with men? |
|  | 5 | Have you ever avoided going to health care services because you worry someone may learn you have sex with men? |
| **General Social Stigma** | 8 | Have you ever felt that the police refused to protect you because you have sex with men? |
|  | 9 | Have you ever felt scared to be in public places because you have sex with men? |
|  | 10 | Have you ever been verbally harassed and felt it was because you have sex with men? |
|  | 12 | Has someone ever physically hurt you (pushed, shoved, slapped, hit, kicked, choked, or otherwise physically hurt you)? [AND] Do you believe any of these experiences of physical violence was/were related to the fact that you have sex with men? |
|  | 13 | Have you ever been forced to have sex when you did not want to (by forced, I mean physically forced, coerced to have sex, or penetrated with an object, when you did not want to)? [AND] Do you believe any of these experiences of sexual violence were related to the fact that you have sex with men? |
| *Note.* Full reliability analyses and factor loadings are previously published^25^ | | |
