# Supplementary material for: Associations between HIV testing and multilevel stigmas among gay men and other men who have sex with men in nine urban centers across the United States
Source: BMC Health Serv Res. 2022 Sep 20;22:1179. doi: 10.1186/s12913-022-08572-4 (PMC9490978; doi:10.1186/s12913-022-08572-4)
Supplement: Supplementary file 2 — Additional file 2: Appendix Table 2. HIV-specific criminalization laws – National HIV Behavioral Surveillance, 9 U.S. sites, 2017. [file 12913_2022_8572_MOESM2_ESM.docx]

| **Appendix Table 2.** HIV-specific criminalization laws – National HIV Behavioral Surveillance, 9 U.S. sites, 2017 | | | | | |
| --- | --- | --- | --- | --- | --- |
| **NHBS Site** | **HIV-Specific Criminal Laws** | **HIV Statute Sentence-Enhancement** | **No HIV Criminalization Laws** | **Felony and/or Misdemeanor** | **Specification for Statute(s)** |
| Baltimore, MD | X | - | - | Misdemeanor | Misdemeanor penalties for engaging in various activities (i.e., consensual sex, sexual crimes, etc.) for knowingly transferring (or attempting to transfer) HIV to another person |
| Denver, CO | - | X | - | Felony | Has HIV sentence-enhancement for people relating to sexual crimes for HIV+ defendants in addition to reckless endangerment |
| Detroit, MI | X | - | - | Felony | Penalties relate to the specific intent to transmit disease in addition to reckless exposure |
| Houston, TX | - | - | X | N/A | No criminal statutes explicitly addressing HIV exposure, but prosecutions have arisen under general criminal laws (i.e., sexual crimes) |
| Long Island/Nassau-Suffolk, NY | - | - | X | Misdemeanor | Does not have HIV-specific criminalization or sentence-enhancement relating to STIs (not specific to HIV), but has misdemeanor charges relating to other offenses (i.e., sexual crimes) for those who are known HIV+ |
| Los Angeles, CA | X | X | - | Both | Has HIV-specific and sentence-enhancement statutes for intent to transmit disease (felony), and disregard for health officer instruction by engaging in activities (i.e., consensual sex, sexual crimes) placing others “at-risk” for HIV transmission (misdemeanor) resulting in increased sentences and mandatory HIV testing |
| Portland, OR | - | - | X | Felony | No criminal statutes explicitly addressing HIV exposure, but prosecutions have arisen under general criminal laws (i.e., sexual crimes) |
| San Diego, CA | X | X | - | Both | Has HIV-specific and sentence-enhancement statutes for intent to transmit disease (felony), and disregard for health officer instruction by engaging activities (i.e., consensual sex, sexual crimes) placing others “at-risk” for HIV transmission (misdemeanor) resulting in increased sentences and mandatory HIV testing |
| Virginia Beach & Norfolk, VA | X | - | - | Both | Has HIV-specific and sentence-enhancement statutes criminalize a broad range of sexual activities for people who are HIV+ and act with the intent to transmit (felony), and must disclose HIV status to partners before engaging in specific oral, vaginal, and/or anal sex (misdemeanor) |
| **Total** | 5 | 3 | 3 |  |  |
| ^1^Material was abstracted from The Center for HIV Law and Policy (2017) | | | | | |
