# Supplementary material for: Associations between HIV testing and multilevel stigmas among gay men and other men who have sex with men in nine urban centers across the United States
Source: BMC Health Serv Res. 2022 Sep 20;22:1179. doi: 10.1186/s12913-022-08572-4 (PMC9490978; doi:10.1186/s12913-022-08572-4)
Supplement: Supplementary file 3 — Additional file 3: Appendix 3 Figure. Conceptual diagram of multivariable generalized hierarchical linear model combining individual and site factors. [file 12913_2022_8572_MOESM3_ESM.docx]

**
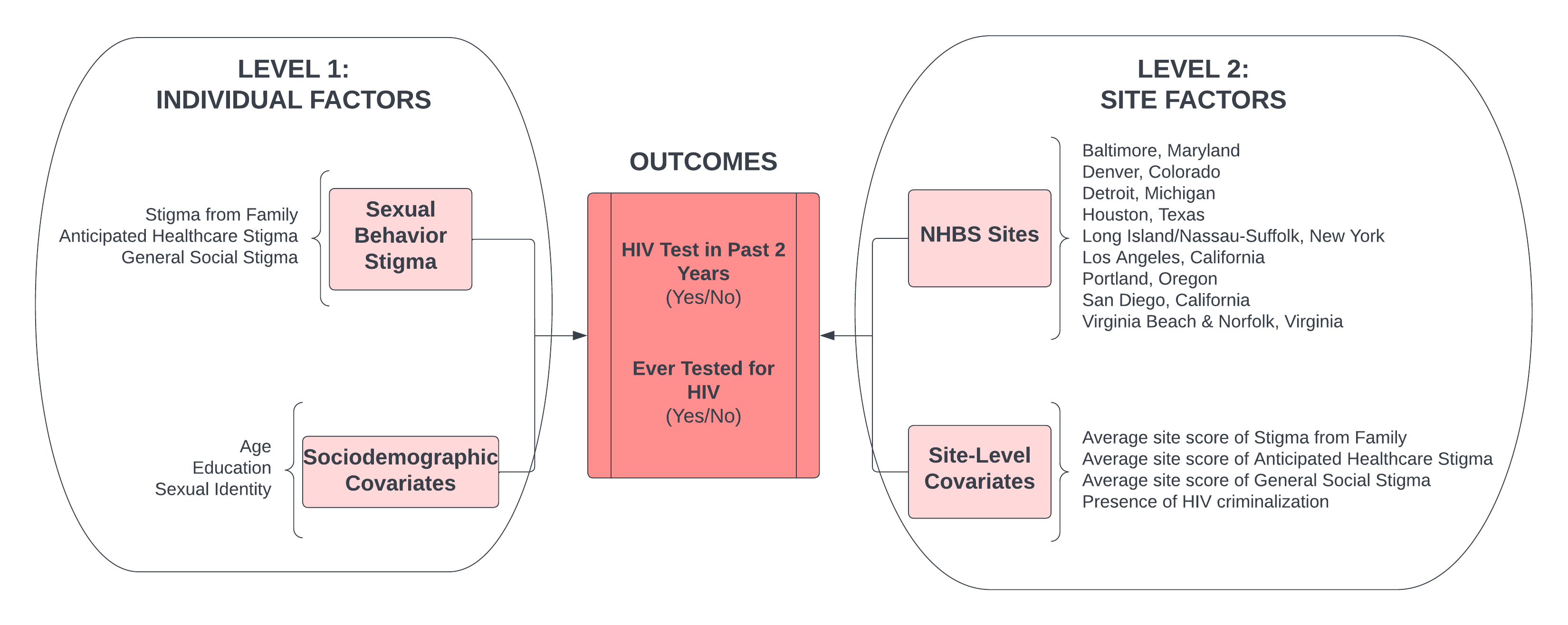
Appendix 3 Figure**. Conceptual diagram of multivariable generalized hierarchical linear model combining individual and site factors
